# Association Between Multinucleated Giant Cells and Recurrence of Anal Fistula: A Prospective Cohort Study

**DOI:** 10.3390/jcm15218518

**Published:** 2026-11-02

**Authors:** Ömer Faruk İnanç, Şermin Çoban Kökten, Kenan Çetin, İsmail Ertuğrul, Wafi Attaallah, Yunus Emre Altuntaş

**Affiliations:** 1 Department of General Surgery, Anadolu Healthcare Center in Affiliation with Johns Hopkins Medicine, 41400 Kocaeli, Turkey; 2 Department of General Surgery, Kartal City Hospital, 34100 Istanbul, Turkey; 3 Department of General Surgery, Faculty of Medicine, Çanakkale Onsekiz Mart University, 17300 Çanakkale, Turkey

**Keywords:** anal fistula, recurrence, giant cell

## Abstract

**Background:** Recurrence of anal fistulas represents one of the most frequently encountered complications in fistula management and remains a dilemma. Studies investigating the histological structure of fistulas and their relationship with recurrence are extremely limited. **Objective:** To evaluate the specimens of anal fistulas for the presence of multinucleated giant cells and to assess whether any correlation existed between giant cell presence and fistula recurrence. **Design:** Prospective cohort study. **Patients:** Adult patients with intersphincteric perianal fistulas included. Patients with recurrent fistulas, Crohn’s disease, tuberculosis, malignancy, immunocompromised status, pregnancy, or those who declined surgical intervention were excluded. A total of 62 patients were included in the final analysis. **Interventions:** Fistulotomy and open biopsy taken from the fistula tract. **Main Outcome Measures:** Postoperative recurrence rates, presence of multinucleated giant cells in the sampled fistula tracts, and the association between giant cell presence and fistula recurrence. **Results:** After 6 months of follow-up, healing was achieved in 52 patients (84%), while fistula persistence was observed in 10 patients (16%) and considered as recurrence. Giant cells were significantly higher in the recurrence group, whereas age, gender, ASA score, and comorbidity did not differ significantly between recurrence and non-recurrence groups. Giant cell presence was observed in 3 patients (5.8%) without recurrence compared to 4 patients (40.0%) with recurrence (*p* = 0.01). Other histological markers showed no significant difference between the groups. **Conclusions:** Our study demonstrates that giant cell presence represents a significant predictor of recurrence in primary cryptoglandular intersphincteric anal fistula.

## 1. Introduction

Anal fistulas represent pathological communications between two epithelial surfaces, specifically establishing aberrant tracts between the anorectal epithelium and perianal cutaneous surfaces [1]. Prevalence of this condition is estimated at approximately 1.7 per 10,000 individuals in the population [2]. Despite considerable advances in diagnostic modalities and therapeutic interventions, anal fistulas continue to pose significant clinical challenges for both patients and surgeons.

The existing literature predominantly focuses on therapeutic modalities for cryptoglandular fistulas, management strategies for secondary etiologic factors, and surgical techniques. Conversely, investigations focused on the histopathological characteristics of cryptoglandular fistulas remain limited.

Recurrence represents one of the most frequently encountered complications in fistula management and remains a dilemma [3,4,5]. Predicting recurrence is not currently feasible. Inadequate surgical techniques, such as operating without identifying the internal opening or the presence of secondary tracts may contribute to recurrence [6,7]. However, studies investigating the histological structure of fistulas and their relationship with recurrence are extremely limited. Occasionally, in patients with non-healing fistulas, Crohn’s disease is diagnosed after a prolonged period, exposing patients to unnecessary repeated surgical interventions [8,9,10]. Since multinucleated giant cells are characteristically observed in Crohn’s disease, we hypothesized that their presence in cryptoglandular anal fistula specimens might be associated with an increased risk of postoperative recurrence. If such an association exists, the presence of these cells may warrant further investigation for underlying Crohn’s disease and consideration of appropriate adjustments in clinical management [11,12].

We evaluated the specimens of cryptoglandular-origin fistulas for the presence of multinucleated giant cells and assessed whether any correlation existed between giant cell presence and fistula recurrence.

## 2. Materials and Methods

### 2.1. Study Design and Setting

This prospective cohort study was conducted at a tertiary colorectal surgery center. A single surgical team to ensure methodological consistency performed all procedures. Patient data were systematically collected using the institutional electronic database system. The study protocol received approval from the Institutional Review Board and was conducted in accordance with the Declaration of Helsinki (Approval number of Ethics Committee: Dr. Lutfi Kırdar Kartal Research and Training Hospital2017/514/120/023). Informed consent was obtained from all participants.

### 2.2. Patient Selection and Classification

Anal fistula classification was based on Parks’ anatomical classification system. Preoperative fistula mapping was performed using anal magnetic resonance imaging for all patients. The study included adult patients (≥18 years) with primary anal intersphincteric perianal fistulas.

Patients with recurrent fistulas, Crohn’s disease, tuberculosis, malignancy, immunocompromised status, or those who declined surgical intervention were excluded from the study.

### 2.3. Surgical Technique and Specimen Collection

All patients underwent surgery in the lithotomy position under general or spinal anesthesia. Fistulotomy was performed, and a tissue specimen was obtained from the fistula tract for histopathological examination. Patients were discharged one day postoperatively and followed up in the outpatient clinic.

### 2.4. Follow-Up Protocol

Patients underwent clinical evaluation at 1 month and 6 months postoperatively. Healing assessment was conducted through clinical examination, with complete healing defined as complete closure of fistula openings and absence of discharge. Persistent fistulas at 6 months postoperatively were classified as recurrence. All patients with persistent complaints were evaluated by anal MRI.

The primary outcome of this study was to evaluate the association between the presence of multinucleated giant cells in fistula tracts and postoperative recurrence. Secondary outcomes included determining the prevalence of multinucleated giant cells and the postoperative recurrence rate.

### 2.5. Histopathological Analysis and Classification

All fistula tract specimens underwent fixation in 10% formalin solution followed by standard tissue processing and paraffin embedding. Serial sections of 4–5 μm thickness were prepared and stained using H&E methodology. Histopathological evaluation was performed using an Olympus BX53 light microscope (Olympus Corporation, Tokyo, Japan). All specimens were assessed by a single experienced pathologist blinded to patient clinical information and outcomes.

**Figure 1:**
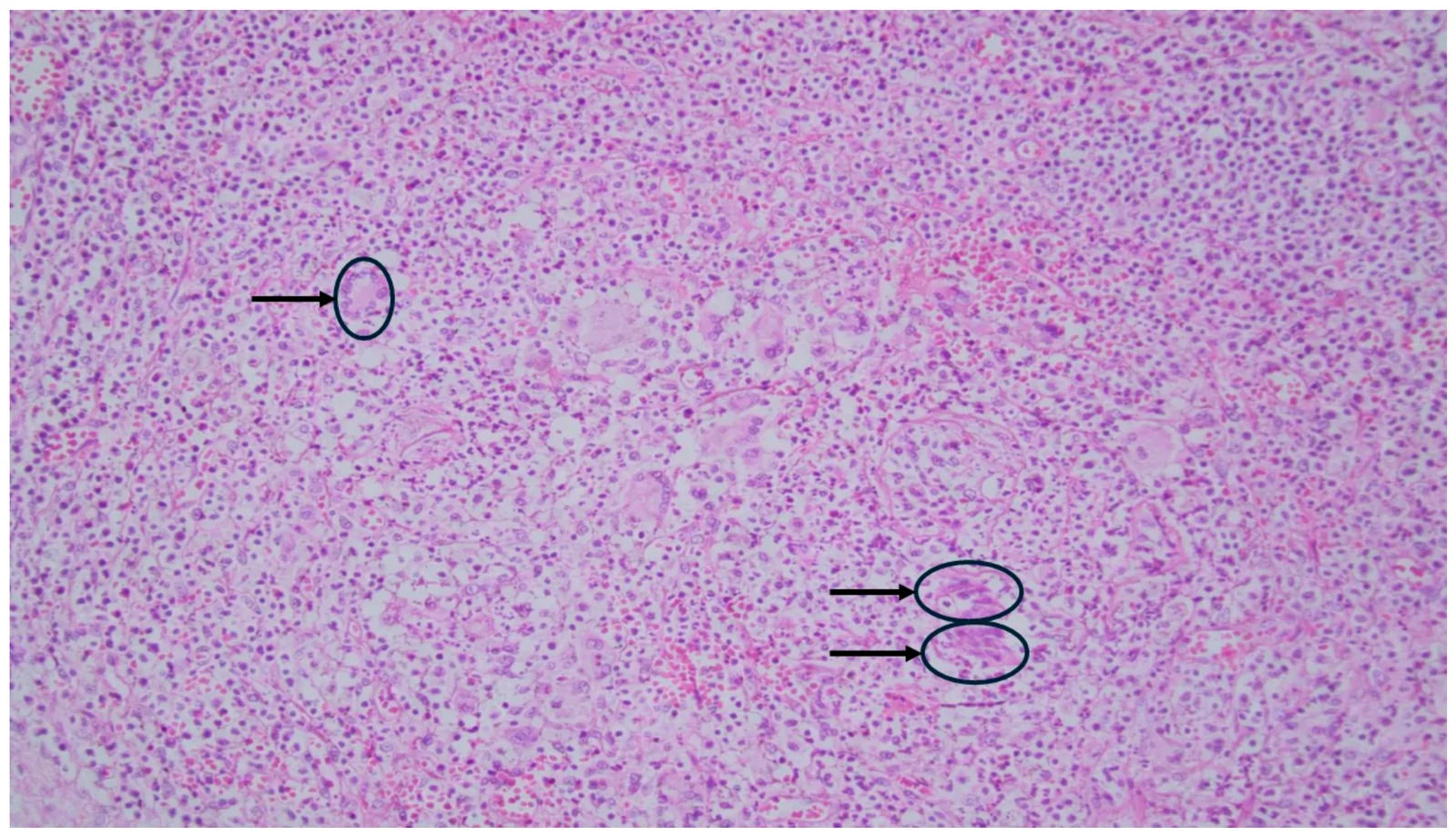
Multinucleated giant cells observed in inflammatory granulation tissue of fistula tract shown by rows (Hemotoksilen & Eosin ×200).

Comprehensive histopathological evaluation was performed at 100× and 400× magnification to assess inflammatory cell populations and plasma cell-to-lymphocyte predominance. The five evaluated cell populations were lymphocytes, plasma cells, histiocytes, granulocytes, and multinucleated giant cells. Lymphocytic infiltration was assessed histologically, and the plasma cell-to-lymphocyte ratio was classified as P > L when plasma cells predominated over lymphocytes. Multinucleated giant cells were assessed qualitatively as a binary variable (present/absent) at 400× magnification. A specimen was classified as positive if at least one multinucleated giant cell was identified in any of the examined microscopic fields; specimens in which no multinucleated giant cells were identified were classified as negative (Figure 1). Histiocytes, granulocytes, and polymorphonuclear leukocytes (PMNLs) were also assessed qualitatively for presence or absence at 400× magnification.

### 2.6. Statistical Analysis

Statistical analyses were performed using IBM SPSS Statistics version 24.0 (IBM Corporation, Armonk, NY, USA). Descriptive statistics were presented as means and standard deviations for normally distributed continuous variables and as frequencies and percentages for categorical variables. Normality was assessed using the Kolmogorov–Smirnov and Shapiro–Wilk tests. Categorical variables were compared using Pearson’s chi-square test or Fisher’s exact test, as appropriate. Normally distributed continuous variables were compared using the independent-samples *t*-test, whereas non-normally distributed continuous variables were analyzed using the Mann–Whitney U test. The Kruskal–Wallis test was used for comparisons involving more than two independent groups. The association between multinucleated giant cell positivity and postoperative fistula outcome was quantified using an odds ratio (OR) with a 95% confidence interval (CI), calculated using an exact method because of the small sample size. All statistical tests were two-sided, and *p* < 0.05 was considered statistically significant.

## 3. Results

A total of 321 patients with anal fistula were evaluated in a single center between January 2018 and January 2019. Among them, 48 patients had anal canal or distal rectal cancer malignancy, 25 had Crohn’s disease-associated fistulas, and 62 had transsphincteric or suprasphincteric fistulas. Of the remaining 186 patients with intersphincteric fistulas, 23 had recurrent disease and were excluded from the study.

**Figure 2:**
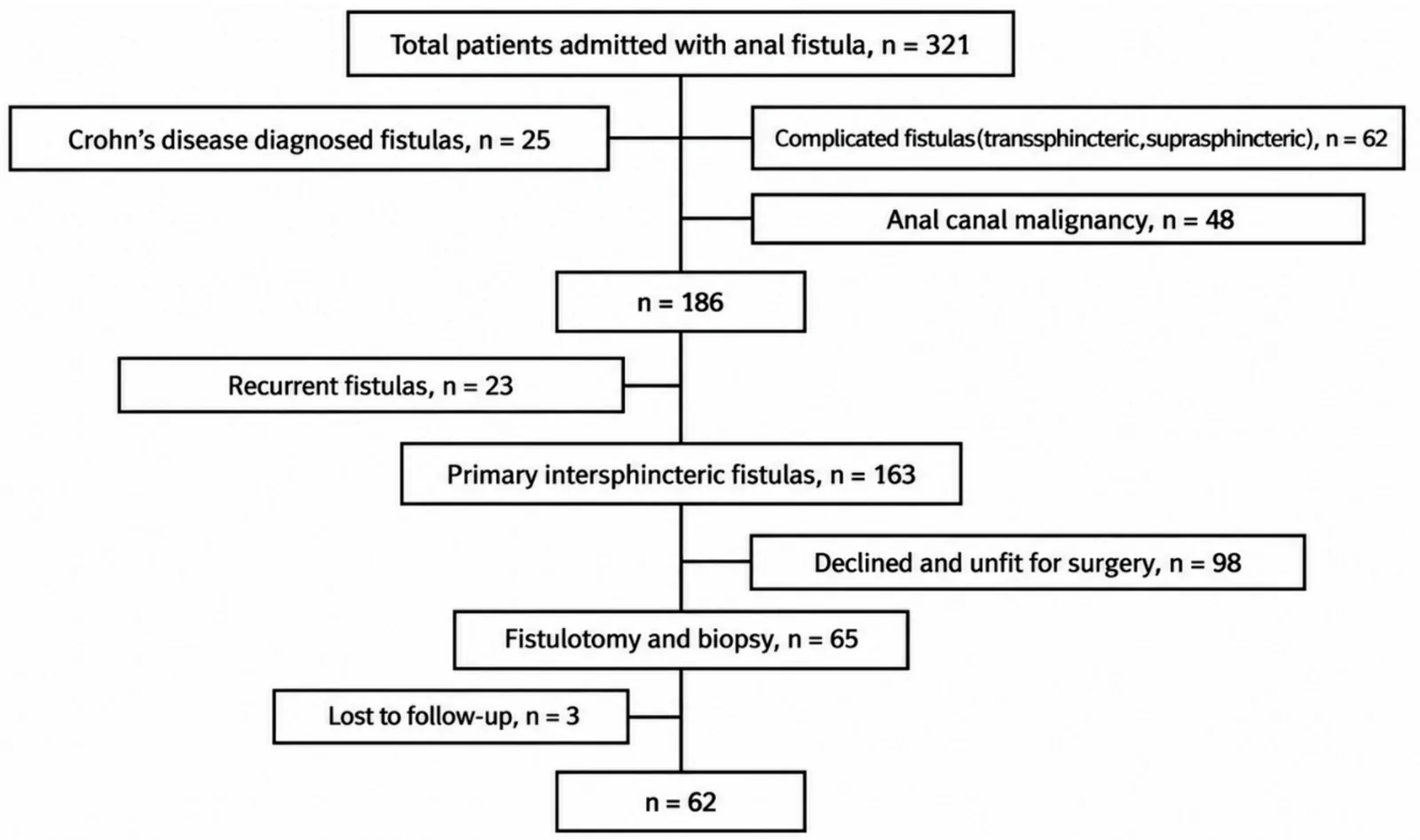
Flowchart of The Study.

Among the remaining 163 patients with primary intersphincteric fistulas who underwent fistulotomy were eligible for the study. The remaining patients (*n* = 98) either declined surgical treatment or were considered unfit for surgery. Three patients were lost to follow-up. A total of 62 patients were included in the final analysis (Figure 2).

### Patient Demographics and Clinical Characteristics

The study cohort comprised 62 patients between January 2018 and January 2019. In total, 49 (79%) patients were male and mean age was age 42.3 ± 13.3 years. American Society of Anesthesiologists (ASA) classification revealed: ASA 1 (*n* = 30, 48%), ASA 2 (*n* = 27, 44%), and ASA 3 (*n* = 5, 8%). No patients were classified as ASA 4.

Comorbidities were present in 11 patients (17.7%), including cardiovascular disorders in 3 patients (27.3%), metabolic disorders in 6 patients (54.5%), and respiratory disorders in 2 patients (18.2%) (Table 1).

After a total of 6 months of follow-up, healing was achieved in 52 patients (84%), whereas recurrence occurred in 10 patients (16%).

Giant cells were significantly higher in the recurrence group, whereas age, gender, ASA score, and comorbidity did not differ significantly between recurrence and non-recurrence group. Other histological markers also showed no significant difference between the groups. Giant cells were detected in 3 patients (5.8%) without recurrence compared to 4 patients (40.0%) with recurrence, demonstrating statistical significance (*p* = 0.01) (Figure 1, Multinucleated Giant Cell). The odds of a persistent or recurrent fistula were higher in patients with multinucleated giant cell positivity (OR, 10.89; 95% CI, 1.95–60.83; Fisher’s exact test, *p* = 0.010).

Polymorphonuclear Leukocytes’ (PMNL) presence was identified in 44 patients (84.6%) without recurrence and 9 patients (90.0%) with recurrence (*p* = 1.00). Lymphoplasmacytic Infiltrates. The distribution of lymphoplasmacytic cell grades showed no significant difference between recurrence and non-recurrence groups (*p* = 0.98). Histiocyte presence was documented in 46 patients (88.5%) without recurrence and 10 patients (100%) with recurrence (*p* = 0.58). Plasma Cell-to-Lymphocyte Ratio (P > L) predominance was observed in 12 patients (23.1%) without recurrence and 4 patients (40.0%) with recurrence (*p* = 0.27) (Table 1).

## 4. Discussion

In this study, a total of 62 patients with intersphincteric anal fistula underwent fistulotomy, and punch biopsy were obtained from the fistula tract during surgery. Multinucleated giant cells were identified in 11% (*n* = 7) of patients. After 6 months of follow-up, recurrence occurred in 16% (*n* = 10) of patients. The detection of multinucleated giant cells was significantly more frequent in the recurrence group than in the healing group.

To the best of our knowledge, this is the first study evaluating the association between multinucleated giant cells and recurrence in primary cryptoglandular anal fistula. Furthermore, in the prospective design, the homogeneous patient cohort are considered the strength of the present study.

Recurrence in anal fistula remains a major clinical challenge, and no reliable predictor of recurrence has yet been established [13,14,15]. Recurrence rates following perianal fistula surgery demonstrate considerable heterogeneity in the literature, ranging from 7% to 50% [16,17].

Anal fistula healing and recurrence have multifactorial pathophysiology, including fistula localization, abscess formation, and patient-related factors such as body mass index, comorbidities, perianal infectious diseases, inflammatory bowel disease, and previous surgical procedures [18,19,20]. However, histopathological characteristics of the fistula tract and their potential relationship with recurrence have received limited attention in the literature.

Although the fistulas in our study cohort were single, simple, and primary intersphincteric fistulas, recurrence was observed in 16% of patients. None of the patients had associated abscesses or a known diagnosis of Crohn’s disease. Most patients were relatively young and had no significant comorbidities. Furthermore, all procedures were performed using a standardized surgical technique after clear identification of both the internal and external openings. These findings suggest that factors other than the established risk determinants may contribute to recurrence.

Other inflammatory cell populations and the plasma cell-to-lymphocyte predominance were also evaluated to explore whether broader histopathological features of the inflammatory response were associated with postoperative recurrence. However, none of these parameters showed a statistically significant difference between the recurrence and non-recurrence groups. These findings suggest that, within our study cohort, the association with recurrence was not observed across the other evaluated histopathological parameters. Nevertheless, the small sample size and limited number of recurrence events warrant caution in interpreting these negative findings.

Isolated perianal fistulas may represent the first manifestation of Crohn’s disease in 5–18% of patients [21]. The incidence of perianal fistula in the general population is approximately 0.3%, whereas it increases to nearly 26% among patients with Crohn’s disease [2,22]. While previous studies have described granulomas in the intestinal mucosa, data regarding histopathological findings within anal fistulas remain limited. Previous studies have reported intestinal granulomas or multinucleated giant cells in approximately 15–58% of patients with Crohn’s disease. However, no study reported the granulomas or Giant cells in the fistula tract itself [21,23,24].

Detection of Giant cells was significantly higher among patients with recurrence in comparison with those without recurrence. Studies regarding anal fistulas in Crohn’s disease showed an accumulation of specific cells such as multinucleated giant cells and granuloma which are macrophages and multinucleated giant cells accumulation [25,26]. In this study we showed that 40% of recurrent fistulas included multinucleated giant cells. Thus, the detection of multinucleated giant cells which the main cells granulomas consist of therefore may predict Crohn’s disease.

In perianal fistulas associated with Crohn’s disease, the frequency of multinucleated giant cells and foreign body-type giant cells varies considerably, and granulomas are absent in the majority of cases [27,28,29]. Lau et al. examined the histopathological features of 124 perianal fistula specimens and identified granulomatous inflammation in only three cases [30]. Therefore, the identification of multinucleated giant cells in cryptoglandular fistulas could be considered a predictor of Crohn’s disease.

In this study, interestingly, approximately 40% of the giant cell-positive patients had multinucleated giant cells located within the fistula wall itself. This finding may support our hypothesis regarding the underlying mechanism of recurrence. Given that granulomatous inflammation and multinucleated giant cells have been described in perianal Crohn’s fistula specimens, we performed further investigations to exclude underlying Crohn’s disease. Furthermore, this achievement may lead to a new concept of diagnosing Crohn’s disease particularly isolated anal presentation of Crohn’s disease by taking biopsy from anal fistula wall. This approach may lead to earlier diagnosis of Crohn’s disease, allowing treatment to be started earlier. It may help prevent unnecessary and repeated surgical procedures and improve the chance of treating the disease before it becomes more complex. Therefore, obtaining biopsy samples from all anal fistulas may be important for differential diagnosis and early diagnosis. Furthermore, such patients may keep in mind not to go for further repeated anal fistula surgery until they are sure of Crohn’s disease.

However, we have no data regarding further treatment of the patients with recurrent fistula and whether after receiving treatment for Crohn’s disease and this is another limitation of the study.

In this study, multinucleated giant cells were identified in approximately 40% of recurrent fistulas. This finding supports the hypothesis that a subset of patients with apparently cryptoglandular recurrent fistulas may represent an early manifestation of an underlying inflammatory bowel disorder. Therefore, the presence of multinucleated giant cells may justify further clinical assessment and careful follow-up in selected patients. However, we have no data regarding subsequent investigations, treatment, or long-term outcomes of these patients, and thus the clinical implications of this finding remain speculative and require further study.

The main limitations of this study are that there is no control group of patients diagnosed with Crohn’s disease and no follow-up protocol for further diagnosed Crohn’s disease. The small number of patients, short follow-up, and inclusion of only intersphincteric fistulas are considered other limitations. Additionally, the exclusion of several potentially eligible patients and the requirement for surgical treatment may have introduced selection bias and limited the generalizability of our findings.

Given the small sample size and the limited number of recurrence events, the present study should be considered exploratory. Although multinucleated giant cells were more frequently observed in patients with recurrence, these findings do not establish their role as a definitive prognostic marker. Larger, independent validation studies are needed to confirm this association and determine its potential clinical utility.

## 5. Conclusions

The pathophysiology of anal fistula recurrence remains poorly understood. In this prospective study, multinucleated giant cells were more frequently detected in patients with recurrent cryptoglandular intersphincteric anal fistulas. These findings should be considered exploratory, and larger, independent validation studies are required to confirm the prognostic value of multinucleated giant cells and establish their potential clinical utility.

## Figures and Tables

**Table 1:** Comparing two groups of recurrence and without recurrence (*n* = 62).

	No Recurrence (*n* = 52)	Recurrence (*n* = 10)	*p*-Value
**Gender (male)**	40 (76%)	8 (80%)	1.00
**Age (Median, min-max)**	42.53	41.50	0.83
**ASA**			0.18
score I/II	25 (48%)/27 (51%)	5 (50%)/3 (30%)	
score III	3 (5%)	2 (20%)	
**Comorbidities**	9 (17%)	2 (20%)	0.57
**Giant Cell Presence**	3 (5.8%)	4 (40.0%)	0.01
**PMNL Presence**	44 (84.6%)	9 (90.0%)	1.00
**Lymphoplasmacytic Cell Grade**			0.98
Grade 1	18 (34.6%)	3 (30.0%)	
Grade 2	22 (42.3%)	4 (40.0%)	
Grade 3	12 (23.1%)	3 (30.0%)	
**Histiocyte Presence**	46 (88.5%)	10 (100%)	0.58
**Plasma Cell/Lymphocyte Ratio**			0.27
P > L	12 (23.1%)	4 (40.0%)	
L ≥ P	40 (76.9%)	6 (60.0%)	

## Data Availability

The datasets generated and analyzed during the current study are available from the corresponding author on reasonable request.
